# Biocatalytic conversion of sunlight and carbon dioxide to solar fuels and chemicals

**DOI:** 10.1039/d2ra00673a

**Published:** 2022-06-06

**Authors:** Mandy Ching Man Yau, Martin Hayes, Shafeer Kalathil

**Affiliations:** Hub for Biotechnology in the Built Environment, Department of Applied Sciences, Faculty of Health and Life Sciences, Northumbria University Newcastle NE1 8ST UK shafeer.kalathil@northumbria.ac.uk; Johnson Matthey Technology Centre Cambridge Science Park, Milton Road Cambridge CB4 0FP UK

## Abstract

This review discusses the progress in the assembly of photosynthetic biohybrid systems using enzymes and microbes as the biocatalysts which are capable of utilising light to reduce carbon dioxide to solar fuels. We begin by outlining natural photosynthesis, an inspired biomachinery to develop artificial photosystems, and the rationale and motivation to advance and introduce biological substrates to create more novel, and efficient, photosystems. The case studies of various approaches to the development of CO_2_-reducing microbial semi-artificial photosystems are also summarised, showcasing a variety of methods for hybrid microbial photosystems and their potential. Finally, approaches to investigate the relatively ambiguous electron transfer mechanisms in such photosystems are discussed through the presentation of spectroscopic techniques, eventually leading to what this will mean for the future of microbial hybrid photosystems.

## Abstract 2

Interfacing photocatalysts with microbes to produce solar fuels and chemicals from carbon dioxide and sunlight.

## Graphical abstract

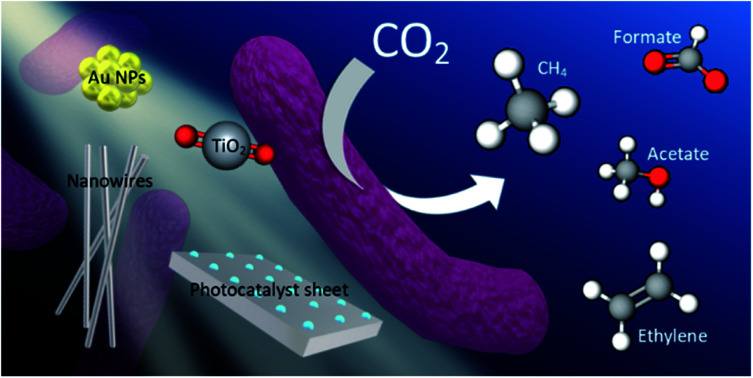

## Introduction

Initiated by the industrial revolution in the eighteenth century, our world has made major strides in several industries such as transport, research and development, and agriculture. However, coupled with the increase in human population, there is now a greater energy demand, consequently generating the current global warming crisis from rising carbon dioxide (CO_2_) emissions. Hydrogen (H_2_) gas, a traditional energy carrier most commonly used in the production of fertilisers and refinement of oil, is a green alternative to reduce carbon emissions.^1–3^ It has a high energy density and a variety of applications but, along with carbon monoxide (CO), it is normally generated through the combustion of fossil fuels. Moreover, the biofuel is limited to H_2_*i.e.*, more complex biofuels such as ethanol and acetate cannot be made. Other current renewable technologies, such as solar and wind power, suffer from low efficiencies due to the fluctuations in the energy source input and this has created a large disparity between the supply and demand.^4–6^

There are several pathways toward the synthesis of green, synthetic fuels to aid in the reduction of carbon emissions. This review aims to explore how biological hybrid systems can be exploited to utilise CO_2_ reduction to produce solar fuels and chemicals. While Reisner's article discusses similar topics in great detail,^7^ this updated review gives precedence to microbial hybrid photosystems and in particular, carbon dioxide fixation. This article also places the global importance of hydrogen as a fuel and touches upon the endeavours in the bioengineering, kinetics and proteomic and metabolomic aspect of microbial hybrid photosystems. Moreover, the potential for these microbial photosystems to be implemented on a commercial scale and how these biotechnologies are being supported is discussed.

Traditionally, artificial photosystems have high solar-to-chemical conversion efficiencies compared to natural photosynthetic systems, but they still have several cons – low stability, high costs, and more importantly, they struggle to produce multicarbon products such as acetate.^8,9^ Microbial photohybrid system, on the other hand, is an emerging green technology and have great promise to overcome those challenges and become a viable alternative to current artificial photosystems.

**Fig. 1:**
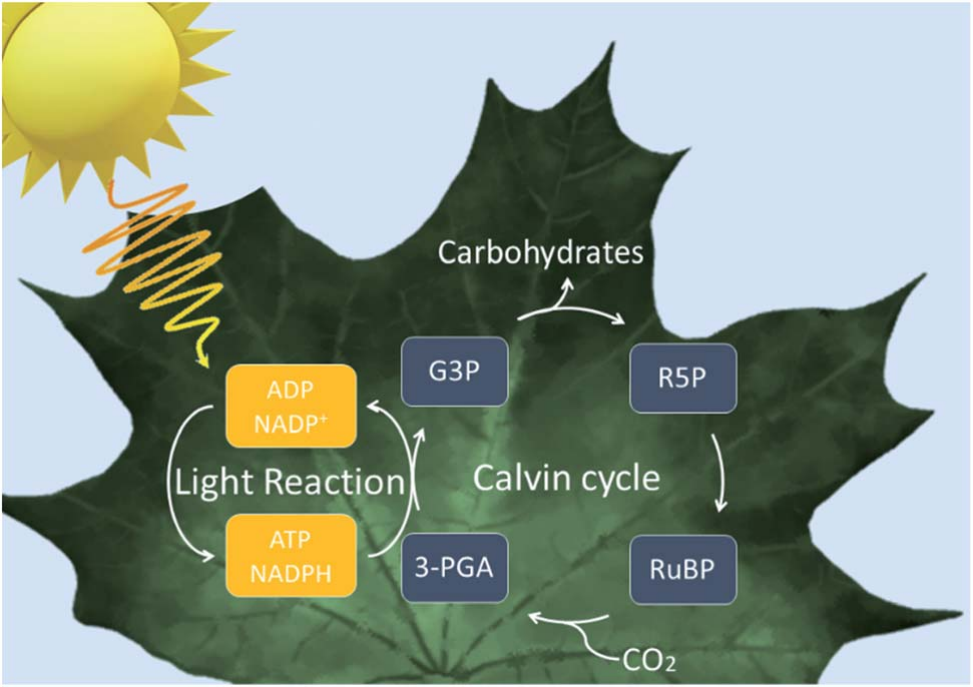
Overview of the reactions within a natural photosynthetic system. Upon light absorption, electrons are excited twice by the photosystems to reduce nicotinamide adenine dinucleotide phosphate (NADP^+^) to NADPH. Adenosine triphosphate (ATP) is also generated upon water oxidation and electron transport which resulted in a proton gradient which drive ATP synthesis. In the dark reaction, CO_2_ (and water) are combined with ribulose-1,5-biphosphate (RuBP) to yield 3-phosphoglycerate which is reduced to glyceraldehyde-3-phosphate with ATP and NADPH.

To first understand how the idea of assimilating biocatalysts with inorganic semiconductors was first conceived, we must look at natural photosynthesis. Photoautotrophs, such as cyanobacteria and plants, utilise photosynthesis where solar energy and CO_2_ are used to produce biomass. There are two phases to this reaction: light-dependent reaction and dark reaction. In the light-dependent reaction, light energy is used to make the reducing equivalents before proceeding to the dark reaction where they convert CO_2_ into carbohydrates and other organic molecules *via* the Calvin cycle (Fig. 1).^10^ The overall efficiency for the conversion of solar energy to biomass is low due to energy losses occurring throughout the system.^11^ However, the quantum efficiencies are high.^12,13^ Therefore, natural photosystems have been used as a blueprint for system design in current artificial and semi-artificial photosystems.

## Artificial photosystems

**Fig. 2:**
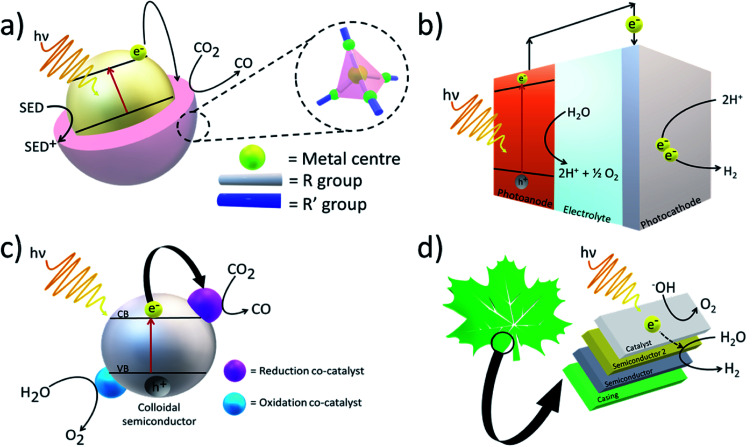
Example artificial photosystems showing (a) a zeolitic imidazolate framework (ZIF) composed of tetrahedrally-coordinated transition metals and imidazolate linkers. ZIFs showing high adsorption and complexation towards CO_2_ are combined with semiconductors with more appropriate bandgaps to generate electron–hole pairs for the photocatalytic reduction of CO_2_. (b) a photoelectrochemical cell producing H_2_ from solar energy (c) a general nanoparticle-based semiconductor for photocatalytic conversion of CO_2_ to CO (d) artificial leaf composed of a tandem construct to produce H_2_ from solar energy.

Artificial photosystems have been commonly used to convert solar energy and feedstock chemicals such as CO_2_ into fuels (Fig. 2). For example, Fujishima and Honda first demonstrated water splitting using solar energy and a photoelectrochemical (PEC) cell consisting of a titanium dioxide (TiO_2_) electrode.^14^ Upon exposure to light, if the photon energy is equal to or greater than the bandgap of the material, electron–hole pairs are generated, and they can either recombine to generate heat or take part in photoreactions. Although PEC cells are theoretically able to achieve fairly high solar-to-chemical (STC) efficiencies, in the case of water oxidation, four electrons and four protons are lost to generate a O–O double bond (498.36 ± 0.17 kJ mol^−1^).^15^ Thus, solar water splitting can be very energetically demanding with high kinetic and thermodynamic barriers. Nevertheless, to achieve high solar water-splitting efficiency, it is essential to develop a proton reduction catalyst capable of catalysing the production of H_2_*via* the conduction band edge potential of the semiconductor.

In comparison, CO_2_ is more thermodynamically stable due to the presence of the strong C=O double bond (532.2 ± 0.4 kJ mol^−1^).^15^ Hence, more amount of energy will be required to overcome the activation barrier. Similar to water splitting, upon light absorption, an electron–hole pair is generated and to favourably reduce CO_2_, the choice of photocatalyst is extremely important. The photocatalyst should contain a suitable band structure where the conduction band edge must be more negative than the redox potential of CO_2_ (−1.9 V *vs.* normal hydrogen electrode at pH 7) and the valence band edge more positive than water oxidation.^16,17^ If not, surplus photon energy will be lost as heat. Currently, there are limited materials with the suitable alignment of the energy band positions. Different strategies to alter the band gap have been employed including doping and surface modifications. Compared to plain bismuth oxide (Bi_2_O_3_), α-Bi_2_O_3_ doped with selenium had a smaller bandgap and increased photocatalytic activity.^18^ In a more novel approach, tuning the stoichiometric amount of formic acid added to a dicyandiamide polymerisation reaction led to the tailoring of the O- and N- link chains in the polymer which determined the band position shifts.^19^

The use of quantum dots (QDs) has also attracted lots of attention due to the quantum confinement effect giving rise to its distinct electronic properties, particularly its size-dependent bandgap.^20^ H_2_, CO, and methane (CH_4_) were all able to be selectively retrieved in the photoreduction of CO_2_ by tuning the size of the cadmium selenium (CdSe) crystals that had been doped onto nickel oxide.^21^ This in turn allowed for the control of the electron transfer kinetics. In another example, a zinc/cobalt-based ZIF was used to coat caesium lead bromide (CsPbBr_3_) QDs to greatly enhance the photocatalytic reduction of CO_2_ into CO and CH_4_.^22^ Compared to bare CsPbBr_3_, the electron consumption rate of CsPbBr_3_-ZIF67 and CsPbBr_3_-ZIF8 was 2.66 and 1.39 greater, respectively. *In situ* photoluminescence measurements on cobalt-containing ZIF revealed its role in promoting electron to cadmium sulfide (CdS).^23^ In other studies, the potential of ZIFs in the activation of CO_2_ has been demonstrated through enhanced activity.^24–27^

Layered double hydroxides (LDH) have previously been investigated for the use of CO_2_ reduction catalysts but the reported efficiencies have been low.^28,29^ By introducing palladium as a co-catalyst, a carbon nitride and LDH assembly proceeded to show an increase in reduction efficiency.^30^ This was due to the distortion of CO_2_ upon interaction with the photocatalyst surface, thus allowing for high reactivity of the photocatalyst for CO_2_ conversion. A similar improvement was also seen in the reduction of CO_2_ to CH_4_ through the use of sodium niobate (NaNbO_3_) nanowires in which higher photocatalytic activity was observed compared to its bulk counterpart.^31^ However, the reason for the increase in photocatalytic activity in this system is most likely due to the surface-to-volume ratio and crystallinity.

Whilst artificial photosystems have greater solar to fuel efficiency compared to natural photosystems, there are still several drawbacks including high costs, toxicity, relatively low efficiencies, and inability to produce higher fuels – all of which are bottlenecks for commercialisation.^6,32–34^ Over the last few years, there has been a shift in focus towards the development of biological-based photosystems, *i.e.*, photobiohybrid systems. Their intrinsic metabolic pathways and numerous enzyme cascades enable the efficient conversion of simple feedstock into complex solar fuels and chemicals with greater product selectivity and stability. Photobiohybrid systems can be classified as enzymatic or microbial according to the biocatalyst.

## Enzymatic photosystems

**Fig. 3:**
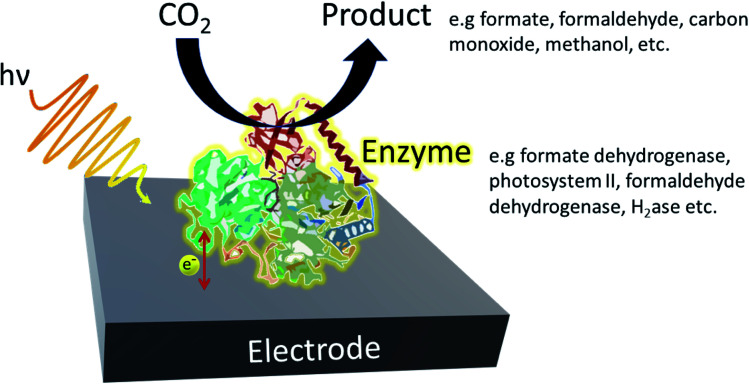
Example of a typical enzymatic photohybrid system in which CO_2_ is reduced by the enzyme to produce the appropriate biofuel.

In a typical enzymatic photosystem, to which artificial photosystems are inspired, photocatalysts use solar energy to convert and generate photoexcited electrons which are then used to convert reducing equivalents or are directly supplied to the enzyme (Fig. 3). The selective nature of enzymes limits the breadth of CO_2_ transformations one can carry out. Therefore, although high rates and yields are achieved, sourcing the most suitable enzyme may result in a rise in costs. Redox enzymes play an important role in industrially relevant bioreactions and to carry out their jobs, reducing equivalents are generally required. Typically, nicotinamide adenine dinucleotide (NADH) and NADPH are generated by a secondary enzyme within the system. However, as mentioned above, photocatalysts can also provide reducing equivalents or directly transfer photoelectrons to active redox reactions. Photohybrid systems utilising enzymes have shown success in not only CO_2_ reduction processes but also nitrogen-fixation and the synthesis of value-added products.^35^

Hydrogenases, a type of metalloenzyme, are often used in the study of H_2_ evolution due to their remarkable catalytic activity. In some cases, turnover frequencies of greater than 1000 s^−1^ have been reported.^36–38^ [FeFe]-based hydrogenase was one of the first reported to be able to receive electrons from photocatalysts such as complexes involving rare metals and carbon nanotubes.^39,40^ Taking [FeFe]-hydrogenase from *Clostridium acetobutylicum*, Brown *et al.* integrated the enzyme with cadmium telluride (CdTe) nanocrystals but the random nature of absorption throughout the complex limited the efficiency of electron transfer complexes. This led to limited H_2_ production.^41^

A common approach to producing formic acid from CO_2_ is through the use of formate dehydrogenase (FDH) as a biocatalyst. Using FDH derived from *Saccharomyces cerevisiae* and zinc tetrakis(4-methylpyridyl)porphyrin as the light harvester, 62 μM formic acid was produced after 3 hours of irradiation.^42^ This was carried out in the presence of methyl viologen (MV) as the electron mediator. On the other hand, Noji *et al.* used tri(bipyridine)ruthenium(ii) (Ru(bpy)_3_^2+^) as the photosensitiser to produce formic acid from CO_2_.^43^ The use of a porous glass plate increased the conversion efficiencies of Ru(bpy)_3_^2+^/MV^2+^/FDH from 1% to 22%. The nanopores within the porous glass plates allowed for the faster accumulation of MV^+^˙ than in the solution, leading to greater interactions between FDH and MV^+^˙ due to the higher density. This is also previously seen when the rate of photoreduction was 16 times higher in a nanopore than in solution when photosystem II was employed as the photocatalyst.^44^

In a hybrid system containing anatase/rutile TiO_2_ mixture as the semiconductor, a ruthenium bipyridyl photosensitiser, and the enzyme carbon monoxide dehydrogenase, CO_2_ was reduced to CO.^45^ However, a SED was required to quench the hole left by the ruthenium complex and to regenerate the photosensitiser. Moreover, the average turnover rate with P25 TiO_2_ was only 0.14 s^−1^ at 20 °C which was lower than with a MV radical (100 s^−1^). Even the addition of the electron donor ethylenediaminetetraacetic acid (EDTA) did not show any noticeable improvement, suggesting inefficient photosensitiser regeneration. A range of surface linkers was also tested to study the effects of enzyme binding to the TiO_2_ surface with regard to electron transfer efficiency. However, those tested, including polymyxin B sulfate and glutamic acid (which had been previously described to strongly coordinate with TiO_2_)^46^ showed very little change in activity and stability. In one case, the addition of *o*-phosphorylethanol amine even resulted in a lower enzyme uptake and thus, decreased the rate of catalysis.

## Microbial photosystems

Enzymatic inorganic hybrid systems appear to overcome the challenges presented by artificial photosystems. However, as described by the above studies, they suffer from several disadvantages including instability issues, poor durability, complicated experimental conditions, and high costs (Table 1). Out of the many carbon fixation pathways known, the Wood–Ljungdahl pathway is a classic for CO_2_ fixation. In this approach, CO_2_ is reduced to acetyl-coenzyme A and eventually converted to acetic acid which can be further used to make more complex products.^47–51^ By utilising the countless metabolic pathways present within the microbe and combining it with the high solar-to-energy efficiencies achieved by artificial semiconductors, products more complex and selective than what enzymes are capable of producing can be generated.

**Table 1:** Summary of advantages and disadvantages of different photosystems

Type of photosystem	Advantages	Disadvantages
Non-biological	High solar energy conversion efficiencies	Low product selectivity from CO_2_ reduction
	Ease of design, tunability, and characterisation	Tends to produce CO only pH gradients result in transfer limitations and may subject the electrodes to corrosion
	Broad absorption bands	Internal resistance of the system may require an external bias to overcome
		Use of limited raw materials *e.g.*, rare earth metals
Enzymatic	High rates and yields	Expensive to isolate and purify
	High selectivity towards CO_2_-to-chemicals	Sensitive to a variety of environmental conditions (*e.g.*, O_2_, pH and temperature)
Microbial	Utilises a myriad of enzyme cascades and metabolic pathways to synthesise complex products from simple feedstocks	Prioritises survival over efficiency
	Resilient to environmental stress	Susceptible to electron losses due to slow kinetics between microbe and electrode
	Self-healing capabilities	ROS produced during photocatalysis may deactivate microbial activities
	Can be genetically engineered to increase conversion efficiencies, produce more complex chemicals, and improve product selectivity	
	Mild reaction conditions	

In the following discussion, we summarise the recent achievements of artificial microbial photohybrid systems utilising solar energy and waste carbon to produce solar fuels and chemicals. Microbial photohybrid systems typically rely on the photosensitisation from organic or inorganic light absorbers to provide the reducing equivalents that allow them to carry out CO_2_ reduction. Nanoparticles, in particular, are especially attractive and have drawn a lot of interest due to their quantum confinement effect. Essentially, if the size of the particle is comparable to that of the Bohr radius, then the optical and electronic properties become size-dependent.^52,53^ By altering the valence and conduction bands of the material, photocatalytic activity can be improved and in turn, greater solar-to-fuel efficiencies can be achieved.

Semiconductors are a vital aspect when striving towards an efficient photosystem capable of converting solar energy to biofuels. When discussing semiconductors, it is natural to immediately think about all those made in the laboratory. Decades of science and technology have made probing into light-harvesting capabilities of countless photosystems possible. However, long before we had the technology to investigate these systems, and before man-made semiconductors came about, nature had its inherent semiconductor found, remarkably, in rocks. In rock samples taken from China, Li *et al.* found that most of the samples had thin iron and manganese oxide coatings.^54^ Presently, researchers often use Mn- and Fe- oxide in the study of photochemical systems due to their efficiency as photocatalysts and their intrinsic properties. As expected, it was found that photocurrent was only detectable in the Fe- and Mn- region on the coating and, upon exposure to solar energy, generated approximately 2.23 × 10^16^ photoelectrons. This is very meaningful as those photoelectrons could act as a major energy source to fuel the growth of photoelectronic bacteria which has been reported numerous times.^55,56^ The study conducted by Li *et al.* is an important precursor to the research into artificial microbial systems as it reinforces the current research on the stimulation of bacterial growth obtained through phototrophic experiments using semiconductors as photocatalysts.^57^

A pivotal development in the world of microbial photosystems was made by Sakimoto *et al.*, in the study of *Moorella thermoacetica* with CdS QDs.^47^ From Tauc plots, a method of estimating bandgap energies, and absorption spectra, the quantum confinement effect was considered to have come into play when the direct bandgap was reported to be 2.51 ± 0.05 eV, as opposed to the expected lower value of 2.42 eV. The electrons generated by CdS when irradiated under visible light generated H^+^, a reducing equivalent, which was then used in the Wood–Ljungdahl pathway to produce acetic acid from CO_2_ with a maximum yield of 90%. Later, TiO_2_-manganese phthalocyanine (MnPc) was added to form a tandem system and cysteine/cystine (CySS/Cys) was chosen as the redox couple.^58^ In general, this expanded hybrid system demonstrated greater acetic acid production. The system containing bare TiO_2_ and *M. thermoacetica*–CdS had greater acetic acid production than *M. thermoacetica*–CdS but less than that of TiO_2_–MnPc. These results demonstrate the superior efficiency of MnPc as a catalyst for CySS reduction. However, a recent study has highlighted pitfalls in the usage of organic chemicals such as cysteine as the SED.^59^ The study revealed that bacteria can convert cysteine to acetate under dark condition which was largely neglected in previous studies due to poor control experiments. Hence, extreme care is needed in the selection of SED as bacteria can metabolize various substrates. Overall, this milestone, along with many other microbial photobiohybrid systems including the use of *Clostridium ljungdahlii* with CdS, *Escherichia coli* with Eosin Y, and *Sporomusa ovata* with silicon nanowires have led to the innovation of many new and improved microbial hybrid photosystems (Table 2).

**Table 2:** Example of hybrid photosystems

System	Biological substance	Light absorber	Reaction	Production rates/yields/activity	Comments	Reference
Inorganic	—	Magnesium–aluminium–LDH/carbon nitride	CO_2_ to CH_4_	3.7 μmol in 24 hours	—	30
	—	NaNbO_3_ nanowire	CO_2_ to CH_4_	653 ppm h^−1^ g^−1^	—	31
		NaNbO_3_ bulk		32 ppm h^−1^ g^−1^	—	
	—	Cobalt-ZIF-CdS	CO_2_ to CO + H_2_	124.4 μmol in 3 hours	TEOA = SED, bipyridine = e^−^ transfer assistant, 82% CO selectivity	23
	—	Lanthanum/rhodium-doped strontium titanium(iii) oxide photosheet	CO_2_ to formate	0.08% solar to formate conversion efficiency	97% formate selectivity	142
Enzymatic	Carbon monoxide dehydrogenase I	Anatase/rutile TiO_2_–RuP	CO_2_ to CO	0.14 s^−1^	2-(*N*-Morpholino)ethanesulfonic acid = SED	45
	Formate dehydrogenase	tetrakis(4-methylpyridyl)porphyrin	CO_2_ to HCOOH	62 μM in 3 hours	TEOA = SED	42
					MV^2+^ = e^−^ mediator	
Microbial	*E. coli*	Silver indium sulfide/indium sulfide	H^+^ to H_2_	1660 μmol in 3 hours	Cysteine = SED	68
	*M. thermoacetica*	Gold nanocluster	CO_2_ to acetate	6.01 mmol g^−1^ over 7 days	Cysteine = SED	67
	*S. ovata*	Silicon nanowires	CO_2_ to acetate	0.3 g L^−1^ over 7 days	—	104
	*R. palustris*	Graphitic carbon nitride	Fructose to PHB	6.73 g L^−1^ after 96 hours	TEOA = SED	69
	*R. eutropha*	CdS nanorods	CO_2_ to PHB	28 mg over 48 hours	10% lactic acid = SED	70
		Graphitic carbon nitride catalyse	CO_2_ to PHB	41.02 mg L^−1^ after 48 hours	—	73
		Core–shell QDs	PHB	100 mg g^−1^ d^−1^	l-Ascorbic acid or HEPES = SED	71
			2,3-Butanediol	10 mg g^−1^ d^−1^		
			Ethylene	1 mg g^−1^ d^−1^		
			Isopropanol	2 mg g^−1^ d^−1^		
	*R. capsulata*	Bi_2_O_3_	H^+^ to H_2_	∼0.26 mL h^−1^	MV^2+^ = e^−^ mediator	80
					EDTA = SED	
	*M. barkeri*	n^+^/p-Si/NiMo	CO_2_ to CH_4_	17.6 mL over 72 hours	H_2_ = e^−^ donor	107
		CdS	CO_2_ to CH_4_	0.19 μmol h^−1^/13.70 μmol	Cysteine = SED	64

As can be inferred from the examples of microbial photobiohybrid systems listed above, there are two approaches in which microbes can be interfaced with synthetic light absorbers: colloidal and non-colloidal. We begin the discussion below with colloidal microbial photosystems before moving on to non-colloidal systems which involve the use of nanowires and electrodes.

## Microbial colloidal photosystems

In a typical colloidal photosystem, the semiconductor is present as a suspension. In some cases, they may be deposited onto the surface of the microbe. Such is the case for the microbial photohybrid system consisting of CdS nanoparticles attached to the surface of *Clostridium autoethanogenum*.^60^ Under light exposure, the CdS–*C. autoethanogenum* system produced almost 4-fold greater acetate than under dark conditions, and a 3-fold increase was observed under CO_2_-only, confirming the H_2_-driven autotrophic conversion to acetate. The system used ethanol to enable the solubility of lipoic acid in the culture medium. Later, it was confirmed that *C. autoethanogenum* oxidised ethanol due to the metabolic shift in response to the change in the redox environment. Ethanol oxidation enabled the hybrid system to obtain NADPH from the oxidised redox environment and this led to greater biomass production. Acetate production and NAD(P)H/NAD(P)^+^ increased when paired with H_2_. NAD(P)H/NAD(P)^+^ play an important role in various biological processes, including biomass and biofuel production and often prevent efficient biotransformation processes. Therefore, by enhancing the production of NAD(P)H/NAD(P)^+^, increased biomass generation will take place. However, microbial systems often contain various intricate biological networks and physiological systems and can self-organise their metabolic system, such that even with bioengineering, manipulation of the NAD(P)H/NAD(P)^+^ production pathway will be met with unexpected barriers.^61–63^ Future research into strategies to increase NADH/NAD^+^ or NADPH/NAD^+^ production will be required to understand and optimise conditions for NADH/NAD^+^ and NADPH/NAD^+^ production for various biotransformation processes. The CdS–*C. autoethanogenum* system demonstrates the viability of coupling microbes with semiconductors, and although further improvements are to be made in terms of electron capture for NADH and NADPH production, the system shows great potential for increased CO_2_-fixing efficiency in nanoparticle-based hybrid photosystems through electron transfer processes.

The use of CdS nanoparticles has also been implemented in a hybrid system with *M. barkeri* which resulted in a CH_4_ production rate of 0.19 μmol h^−1^ with 0.34% quantum efficiency.^64^ This system was further improved when doped with nickel, as well as CdS, which increased the photoelectron transfer within the system and led to an approximate rate of 0.24 μmol h^−1^ CH_4_.^65^ Investigation into the proteomics of the system revealed that the addition of nickel caused a higher expression of proteins related to electron transfer, energy conversion and CO_2_ fixation which contributed to the increased photoelectron transfer rate. Increasing protein expression is one method to increase electron transfer rates. Another viable method is through pi conjugation. Using the organic semiconductor poly(fluorene-*co*-phenylene) and perylene diimide derivative to photosensitise *M. thermoacetica*, the production of acetic acid from CO_2_ was successful.^66^ Efficient electron transfer was enabled through the pi conjugation present in the semiconductor which has been suggested to exhibit higher biocompatibility. Moreover, the quantum efficiency of the perylene diimide derivative was found to be comparable to inorganic semiconductors.

The production of acetic acid *via* CO_2_ reduction was also realised through the use of gold nanoclusters with *M. thermoacetica*.^67^ In a previous study carried out by Jiang *et al.*, the microbe viability before and after the photocatalytic reaction was measured through cell density and the lack of change indicated good viability of the cells.^68^ In this study, the gold nanoclusters quenched reactive oxygen species (ROS) as well as acted as a light absorber. This enabled *M. thermoacetica* to ensure high bacterium viability over 6 days of continuous CO_2_-fixation, showcasing the system's promising potential as a sustainable system.

Semi-artificial microbial systems can also be used to make more complex biofuels. Proof-of-concept was demonstrated in the works of Xu and coworkers where graphitic nitride and *R. eutropha* H16 were used to increase polyhydroxybutyrate (PHB) production from fructose 1.4-fold with triethanolamine (TEOA) as an SED.^69^ Further works by the group include the use of CdS nanorods to produce PHB from CO_2_ and fructose by *C. necator*.^70^ Optimisation of CdS nanorods resulted in high photoactivity and achieved a yield of 28 mg over 48 hours PHB from CO_2_. On the other hand, Ding *et al.* employed different core–shell QDs to bind to specific enzyme binding sites in bacteria to improve product selectivity in photosynthetic biohybrid systems.^71^ Through this, not only was gram scale production of PHB achieved but also multi-carbon compounds. The production of PHB successfully increased using only a bioreactor with LED panels, illustrating the ease of scaling up beyond laboratory conditions and implementation on a commercial scale.

Many of the hybrid systems mentioned are limited by anaerobic conditions which may impede the assimilation of microbial hybrid photosystems in industry. The hybrid system presented by Liu *et al.* consists of CdS nanoparticles biologically precipitated onto the surface of *Thiobacillus thioparus* and is able to reduce CO_2_ to multicarbon glutamate synthase without being restricted by the need for an anaerobic environment.^72^ Interestingly, while chemically synthesised CdS predictably led to cell death in the later stages of cultivation, the biologically synthesised CdS showed a higher promoting effect for the growth of *T. thioparus*. For the system to sustain cell growth in the presence of biologically synthesised CdS, not only is the electron transfer between the semiconductor-microbe interface efficient but also CO_2_ fixation. Once CO_2_ has been fixed by NADPH, the resulting precursors which undergo chemical transformations within *T. thioparus* become biochemical components that support cell growth and replication. Hence, enabling the hybrid system to undergo self-replication. In not being limited by strict anaerobic conditions and its ability to sustain microbial growth with only simple, economical components, this work shows potential for the reduction of CO_2_ in terms of design and sustainability.

While enzymes and microbes have individually been interfaced with light absorbers, the hybrid system established by Tremblay *et al.* involves both *R. eutropha* and H_2_O_2_-degrading catalyse for bioplastic production and water-splitting, respectively.^73^ Although using the light absorber, graphitic nitride, and *R. eutropha* alone can successfully produce PHB, the yield was lower than with the addition of H_2_O_2_-degrading catalyse. Possible explanations for the increased yield include increased H_2_ for *R. eutropha* to maintain its activity and the use of O_2_ produced from water-splitting as an additional electron acceptor. The increased PHB production resulting from coupling water-splitting reactions and biochemical cascades within *R. eutropha* showcases a promising platform for efficient PHB production. However, compromises may have to be made with regards to performance and design due to the expense and temperament of enzymes.

H_2_ is becoming an increasingly popular energy carrier, as evident by the global initiatives to invest in the hydrogen economy, such as the UK's Hydrogen Strategy,^74^ USA's Hydrogen Program Plan,^75^ Canada's Hydrogen Strategy,^76^ Japan's Basic Hydrogen Strategy,^77^ and the European Clean Hydrogen Alliance which consists of industries and authorities from various countries.^78^ At present, the production of H_2_ is still mainly from fossil fuels but in recent years, research into the electrolysis of water from renewable electricity has garnered a lot of attention. The downfall of this method, however, is that rare and precious metals are used in the synthesis of the catalysts involved, meaning an increase in production costs and limited sustainability and scale-up potential.

As previously stated, current inorganic systems involving photovoltaic technology and semiconductors have surpassed natural biotransformation efficiencies. However, reaching similar conversion efficiencies is still a challenge yet to be achieved. *Clostridium butyricum* and *Escherichia coli*, along with semiconductors, were initially used to explore photocatalytic H_2_ production.^79–84^ To this day, the pool of microorganisms used to explore H_2_ production has expanded to include *Shewanella oneidensis* and more.^85–89^ In the works of Martins and coworkers, *Desulfovibrio desulfuricans*, *Citrobacter freundii*, and *S. oneidensis* were used in conjunction with CdS nanoparticles to investigate H_2_ production.^84^*D. desulfuricans* was found to be the most efficient, owing to the high biological activity and/or efficient electron transfer. This was accompanied by the fact that it showed the highest hydrogenase activity compared to *S. oneidensis*, *E. coli*, and *C. freundii* (280 μmol g_dcw_^−1^ min^−1^, 8.4 μmol g_dcw_^−1^ min^−1^, 3.7 μmol g_dcw_^−1^ min^−1^, and 1.6 μmol g_dcw_^−1^ min^−1^ respectively). High levels of the enzyme hydrogenase correlate with greater H_2_ production as they catalyse H_2_ formation from protons or the oxidation to protons. The high levels of hydrogenase in *D. desulfuricans* were found to be from the [NiFe] and [FeFe] families. These enzymes tend to be located in the periplasm of the microorganism,^90,91^ leading to a more efficient transfer of electrons from the nanoparticle to the enzyme than those found in the intracellular compartment. Cyanide was used to inhibit the hydrogenases within *D. desulfuricans*, almost completely preventing H_2_ formation. This ultimately highlights the importance of hydrogenase in the pursuit of efficient H_2_ formation. H_2_ evolution was also possible through the use of a tandem involving silver indium sulfide/indium sulfide and *E. coli*.^68^ In this system, the quantum efficiency of 3.3% was achieved and showed very little change in cell viability after the photocatalytic reaction, demonstrating the stability of the hybrid system.

A major bottleneck of microbial hybrid photosystems is the photoelectron transfer from the inorganic component of the system to the biological cell. In a study to increase H_2_ production, reduced graphene oxide was chosen to be integrated with *S. oneidensis* MR-1.^92^ The reduced graphene oxide acted as a solid support surface and was able to efficiently collect electrons for the semiconductor, cuprous oxide (Cu_2_O). In addition, the reduced graphene oxide operated as a pathway for electrons to travel between. All the above allowed for efficient electron transfer from the Cu_2_O nanoparticles to *S. oneidensis* MR-1, thus contributing to efficient H_2_ production, as evident in the increased photocatalytic H_2_ production when compared to pristine Cu_2_O and Cu_2_O coupled with reduced graphene oxide (322.0 μmol g_Cu_2_O_^−1^*vs.* 7.0 μmol g_Cu_2_O_^−1^ and 4.0 μmol g_Cu_2_O_^−1^ within 4 hours respectively).

It is important to elucidate the electron transfer pathway to understand the mechanism behind H_2_ evolution within a hybrid system. Upon deletion of H_2_ase, it was clear that within *S. oneidensis*, H_2_ase was the active site for H_2_ evolution as there was no H_2_ production.^92^ Deletion of the proteins MtrC/OmcA was found to have inhibited the production of H_2_, thus establishing the fact that the cytochromes, including MtrA and MtrB, are involved in the electron transfer between the nanoparticle and *S. oneidensis*. While the work by Shen and coworkers concerns H_2_ production, the understanding of the electron transfer pathway in the photocatalytic conversion extends to the production of all biofuels, not just H_2_. In resolving and understanding the electron transfer pathway within a system, the overall design of hybrid systems can then be further improved, yielding greater electron transfer efficiency and ultimately, greater yields.

The kinetics of the electron transfer is extremely important in the conversion of solar energy to solar fuels. The previously mentioned work by Martin and coworkers on *D. sulfuricans* involved hydrogenases found in the periplasm of the microbe. Similarly, *S. oneidensis* also expresses periplasmic hydrogenases.^93^ This was exploited by Shi and coworkers, in which copper indium sulfide/zinc sulfide (CuInS_2_/ZnS) QDs were translocated into *S. oneidensis*, resulting in an increase in H_2_ production due to the decrease in electron transfer distance and energy loss.^94^ Through the deletion of certain genes, and the introduction of MV as a redox mediator, direct electron transfer from the QDs to *S. oneidensis* was inferred. As expected, single and double deletion of the periplasmic hydrogenase, [FeFe] and [NiFe], caused a decrease in the production of H_2_, which suggests the two coenzymes play a crucial role in H_2_ production. Deletion of the electron transport proteins had little to no effect on the production of H_2_, nor did the introduction of MV as a redox mediator.

Manipulation of the genetic material within the microbe is becoming an increasingly popular method of increasing the production of biofuels. Using genetically engineered *E. coli* with precipitated CdS, [NiFe]-hydrogenase HyaABCDEF plasmid was transformed into the bacteria to catalyse H_2_ formation.^95^ Compared to non-HydA-induced hybrids, the resulting output per cell was much greater. In the case involving CdS and *E. coli*, genetic engineering was used to emphasise the expression of [FeFe]-hydrogenase gene, consequently yielding a photoactive *E. coli* hybrid system capable of converting solar energy into H_2_ within a single cell.^96^ Although the yield was less than previously reported studies involving TiO_2_ and *E. coli*,^97^ the genetically engineered hybrid is a viable method for enhancing photocatalytic H_2_ production.

## Microbial non-colloidal photosystems

Along with colloidal systems, microbes have also been interfaced with nanowires and PEC systems. Due to the large surface area, nanowires are extraordinary light harvesters. Initial studies on mammalian cells have demonstrated the ability to recognise and respond to nanoscale topographies.^98–100^ In response to this, a silicon nanowire array was interfaced with *S. oneidensis* MR-1.^101^ Through this model, it was discovered that not only attachment locations were influenced by the topography, but also the swimming patterns of the microbes. In a previous study conducted by Goto *et al.*, the tendency of single flagellated bacteria to travel in a straight trajectory was revealed.^102^ However, *S. oneidensis* was shown to have circled the nanowire despite being a singly flagellated bacterium. Moreover, the reversible bacterial attachment was demonstrated after some of the bacteria were found to separate from the Si array following a short period. The swimming behaviour exhibited by *S. oneidensis* is also observed by *E. coli* K-12 strains.^103^ By highlighting the important role of topography and its interaction with bacteria, knowledge that will serve to aid in the design of future microbial-nanowire interfaces was realised.

As expected from the above study by Jeong *et al.*, the use of a nanowire array proved to be more advantageous than that of a single nanowire.^101^ The use of arrays has been widely implemented in several research studies. Liu *et al.* cultured *Sporomusa ovata* with a silicon nanowire array passivated by TiO_2_ protection layer, leading to the production of acetic acid from CO_2_ after the incubation period.^57^ Using dual light-absorbers allow the system to fully absorb solar energy, thus increasing photovoltage for the system. This is demonstrated by the high faradaic efficiency (90%). This system was taken further and extended by allowing genetically engineered *E. coli* to activate acetate into acetyl-CoA, ultimately partaking in the biosynthesis of various complex molecules such as cadinene, PHB, and *n*-butanol. The study is especially important as the system itself generated a local anaerobic environment at the bottom of the nanowire array, encouraging the reduction of CO_2_ under aerobic (21% O_2_) conditions. Su *et al.* later used highly doped p^+^ silicon nanowire arrays and platinum wire as the counter electrode to replace the TiO_2_ photoanode previously used.^104^ By adjusting the electrolyte pH and increasing the buffering capacity, the group successfully achieved the formation of closed-pack silicon nanowires. The resulting close-packed biohybrid system obtained a solar-to-acetate efficiency of 3.8% and a current density of 0.65 ± 0.11 mA cm^−2^, as opposed to the energy conversion of 0.38% for acetic acid production and 0.35 mA cm^−2^ photocurrent by Liu and colleagues.^57^

**Fig. 4:**
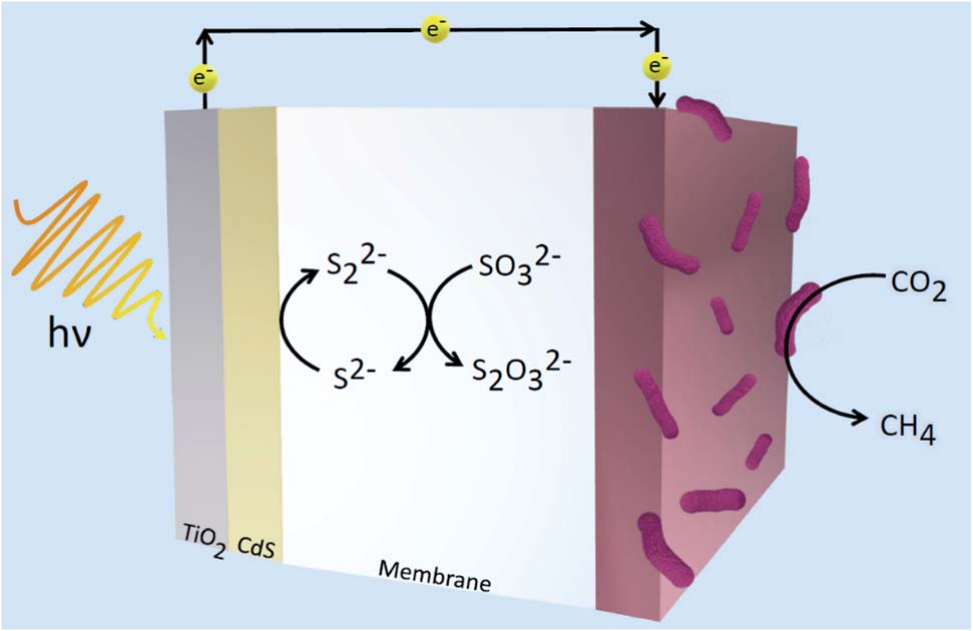
Hybrid microbial photosystem designed by Xiao *et al.* consisting of a TiO_2_/CdS photoanode and a biocathode for CO_2_ reduction to CH_4_.^106^ The sulfide electrolyte solution was used as sacrificial reagents to consume holes and prevent the corrosion of CdS.

Using methanogens in a PEC cell setup, a one-step transformation from CO_2_ to CH_4_ with a faradaic efficiency of up to 96% with only solar energy as the only energy input was achieved.^105^ Solar-to-fuel conversion efficiency was found to be at 0.1%, approximately half of that of natural photosynthesis. The greatest limitation in the system is expected to be from the bandgap of the TiO_2_ photoanode employed. TiO_2_ is often used in designing photosystems, but its downfall is that it can only absorb a small range of wavelengths of solar energy. Thus, in expanding the absorption range of materials used in photosystems, the efficiency will likely increase. A combination of TiO_2_/CdS was also used as a photoanode in the reduction of CO_2_ by methanogens.^106^Fig. 4 illustrates the system constructed by Xiao and coworkers which demonstrated 94.4% faradaic efficiency and solar-to-fuel conversion efficiency of 1.28%.^106^ Cu_2_ZnSn_4_ was later employed to sensitise the photoanode. Due to the lower charge separation resistance, the photogenerated electron–hole separation was much more favoured and this led to greater CH_4_ production. Suspending microorganisms in solution is also a viable method to catalyse redox reactions fuelled by solar energy. By interfacing the archaea *Methanosarcina barkeri* with a platinum electrode, Nichols *et al.* achieved a faradaic efficiency of up to 86% for electrochemical CO_2_ to CH_4_ conversion.^107^ Replacing the platinum electrode with synthetic α-nickel sulfide H_2_ evolution reaction catalyst resulted in similar faradaic efficiencies and CH_4_ yields. Furthermore, by using an indium phosphide photocathode and a TiO_2_ photoanode, the system proceeded to result in unassisted light-driven CH_4_ production from CO_2_ and this system yielded much greater CH_4_ than that with the platinum electrode and α-NiS catalyst.^107^ This proof-of-concept of bridging semiconductors with microbes proves efficient solar conversion to carbon-based fuels is attainable.

## Elucidation of electron transfer mechanisms and microbial life in microbial photosystems

**Fig. 5:**
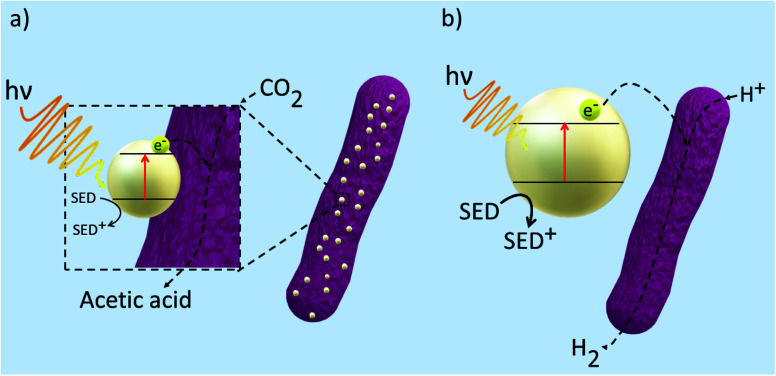
General mechanism for electron transfer between a semiconductor and microbe in a hybrid photosystem. (a) Direct photoreduction of CO_2_ to acetic acid in which the semiconductor is deposited onto the surface of the microbe (b) indirect photoreduction of CO_2_ to H_2_ by colloidal nanoparticles in the presence of electron mediators and SEDs.

Although microbial hybrid systems have proven to be a promising approach to producing biofuels, achieving yields that can compete with current energy outputs from fossil fuels are still yet to be realised. Fig. 5 displays the general mechanisms taking place within a microbial hybrid photosystem but because of the knowledge gaps behind the mechanics of the hybrid system, the finer details are still relatively unknown. Some challenges involve the seamless integration of synthetic and biological products and increasing the efficiencies of light absorption and solar-to-biofuel conversion. To work towards a system that can be applied to the industry, it is important to investigate the electron transfer mechanisms between the inorganic substrate and the biological component as they are the major bottleneck in the system. Therefore, at present, many techniques have been employed to probe into such interactions within the hybrid system to allow us to gain a deeper understanding.

**Fig. 6:**
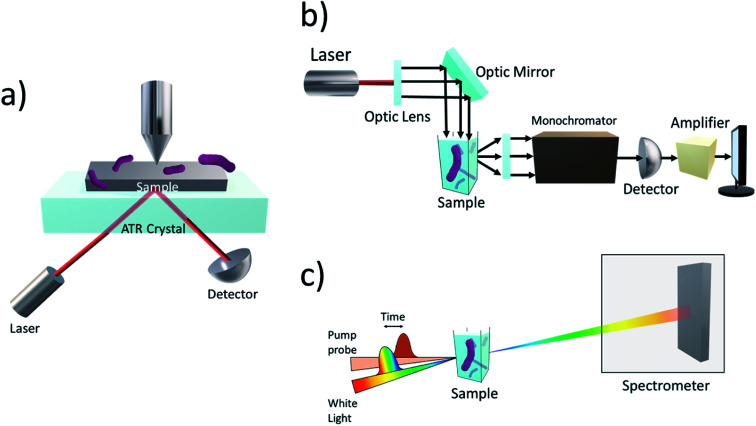
(a) Schematics of ATR. The infrared beam interacts with the biofilm sample at one point of reflection (or at multiple points with multi-bounce ATR). Some of the evanescent waves formed from total internal reflection interact with the sample, resulting in an attenuated total reflection. Reactions can be monitored in real-time and information about kinetics and key transient reaction intermediates can be obtained, aiding in the study of kinetics and mechanism of the system. (b) Experimental setup of photoluminescence spectroscopy. The laser is directed to the sample using optical lenses. Once the laser hits the sample, the electrons are excited to higher energy states and as they relax, radiation is emitted which is detected by the photoluminescence spectrometer at specific wavelengths which reflect the energy level differences. The decay kinetics of the photoluminescent curve obtain will give information about the processes taking place in the hybrid photosystem. (c) In transient absorption spectroscopy, a pump pulse is used to excite the sample containing microbes interfaced with nanoparticles. Changes in the optical absorption of the sample are measured as a function of time and thus, the sample's absorbance is measured before and after pumping. The resulting information regarding the system's decay and lifetimes will shed light onto the kinetics of the system.

Attenuated total reflection infrared (ATR-IR) (Fig. 6a) has been used to probe into the attachment of microbes onto inorganic surfaces in several previous works.^108–111^ ATR-IR spectroscopy is an attractive method for being not only suitable for solid samples but also for biological liquid samples. In the works of Freitag and colleagues, an acoustic trap was combined with ATR-IR spectroscopy to enable the entrapment of enzyme-labelled beads without the need for mechanical retention components, such as optical fibres, which are normally found in typical bead injection systems.^112^ The set-up allowed for the conversion of *p*-nitrophenylphosphate into *p*-nitrophenol and phosphate under varying loadings of enzymes to be monitored, showcasing its great potential for enzymatic kinetic studies.

Steady-state photoluminescence (Fig. 6b) on the previously discussed work by Shen *et al.* was used to investigate the separation and transfer of photogenerated charge carriers in the system.^92^ In comparison with the absence of the Cu_2_O nanoparticle, the steady-state photoluminescence intensity of Cu_2_O/reduced graphene oxide decreased. This indicates that the separation of the photogenerated electron–hole pair was sufficient for a pathway between the Cu_2_O nanoparticle and *S. oneidensis* cells for electron transfer. Similar observations were made with CuInS_2_/ZnS QDs interfaced with genetically engineered *S. oneidensis*.^94^

A more commonly used technique is time-resolved infrared spectroscopy (TRIR). TRIR is often used to study the kinetics, formation of intermediate products, and intramolecular charge transport of both biological and chemical molecular species.^113–115^ For example, TRIR spectroscopy, coupled with transient absorption (TA) spectroscopy (Fig. 6c), was used in an *M. thermoacetica*–CdS hybrid system to explain the decreasing CO_2_ to acetic acid conversion rates with increasing H_2_ incubation in the first three hours of photosynthesis.^116^ Changes in the vibrational modes of CO and carbon nitride, along with other amino acid residues, were observed and due to the peaks decaying on the same timescale as the TA signal, it was speculated the picosecond e^−^ transfer was from a molecular process, not from a purely physical source.

In the same model, TA was used to aid in the elucidation of the decay kinetics of *M. thermoacetica*–CdS. It was reported that *M. thermoacetica*–CdS incubated with H_2_ : CO_2_ displayed faster decay kinetics compared to that incubated with glucose.^47,116^ The faster decay kinetics was attributed to the increased H_2_ase activity and hence, the greater e^−^ transfer rates and/or the greater quantity of e^−^ acceptors in the H_2_ incubated system. Unsurprisingly, multi-exponential decay in the timescale of picoseconds was found to be expressed in the hybrid system. Likewise, in the previously mentioned works of Luo *et al.*, when CuInS_2_/ZnS QDs were integrated with *S. oneidensis* MR-1 through co-incubation, transient fluorescence and transient absorption lifetimes were shorter than that with CuInS_2_/ZnS than with the hybrid system, indicating enzymes are efficient acceptors of photogenerated charge.^94^ Decay kinetics in the same timescale as the *M. thermoacetica*–CdS hybrid was reported in a hybrid metal–semiconductor nanoparticle system consisting of CdSe–Au.^117^ However, lifetimes reported in the range of nanoseconds were found in Fe–Fe H_2_ase–CdS systems,^118^ possibly owing to hydrogen bonding, solvent effects, and charge transfer barriers. From the evidence gathered *via* TA and TRIR spectroscopy, two proposed pathways for charge and energy transfer were proposed: the non-H_2_ase-mediated pathway in glucose incubated cells and the membrane-bound H_2_ase mediated pathways in H_2_ incubated cells. Overall, the existence of a charge-transfer pathway was supported with TA and TRIR spectroscopy.

Due to the extremely fast nature of the charge separation and transfer, (from milliseconds to picoseconds), TA is also capable of providing information on parameters such as triplet excitons, exciton energy, and decay kinetics which can be used to speculate charge transfer processes.^119–121^ The previously mentioned system consisting of cadmium selenium–gold (CdSe–Au) had undergone photoexcitation of the local surface plasmon resonance of the metal. By using ultrafast spectroscopy, along with high temporal resolution and near-infrared excitation, the plasmon-induced charge transfer (PICT) pathway was resolved, and it was discovered that hot-electron transfer to CdSe occurred in the timescale of fewer than 30 femtoseconds, whereas back transfer from the semiconductor to the metal was found to be within 210 fs.^117^ To compete with the extremely fast back-transfer to the metal, extraction of the electrons needs to be greater than the rate of back-transfer. This issue needs to be realised when designing hybrid systems to avoid inefficient and undesired transfer pathways. With suitable band alignments, such issues may be mitigated.

The kinetic study of this hybrid construct using high time resolution TA, along with the aforementioned studies, has highlighted the importance of spectroscopic techniques in the pursuit of elucidating electron transfer kinetics in photoactive systems. In establishing the requirements for resolving ultrafast photophysics of hybrid systems, there will be a greater understanding of the kinetic pathways within a system, resulting in a more intelligent design of hybrid systems which will allow for more diverse photocatalytic applications.

The involvement of proteomic and metabolomic analysis in the study of hybrid systems has recently become more prominent. Whilst the above-mentioned spectroscopic techniques will help clarify kinetics and electron transfer processes, proteomics and metabolomics will enable a greater understanding of the biocatalytic mechanisms, energy conservation in microbes, and physiological state of the organism. Taking the model *M. thermoacetica*–CdS hybrid system developed by Sakimoto and coworkers, Zhang *et al.* studied the energy conservation processes within the system using untargeted and targeted protein and metabolite quantification processes.^122^ Through this approach, it was discovered that the energy-metabolism-associated glycolysis and tricarboxylic acid cycle were active alongside the well-known Wood–Ljungdahl pathway. *M. thermoacetica* was also implied to contain an increased expression of proteins capable of protecting *M. thermoacetica* from oxidative stress but the damage far outweighed the protection provided.

Although the current biohybrid photosystems are still in their infancy, with the study presented by Zhang *et al.*,^122^ the aforementioned spectroscopic techniques, and more importantly, with technological advancements, the means to fabricate highly efficient, low cost, and industrially competitive biohybrid photosystems will become more tangible in the nearby future.

## Conclusion

To conclude, while artificial photosystems and enzymatic photosystems have many advantages, artificial microbial photosystems present themselves as a much more powerful tool for converting sunlight and CO_2_ to biofuels. However, there are still significant developments to be made. The commercialisation of hybrid photosystems continuously proves to be a challenge and several considerations including, but not limited to, cost, toxicity, environmental impact, and the transition from laboratory to industrial scale still need to be addressed further.

With emerging technologies able to elucidate the electron transfer mechanisms in microbial hybrid photosystems, our understanding of the interactions occurring within the system will deepen, allowing for maximisation of solar-to-chemical yields, more complex solar fuel production, and smarter system design which will pave the way towards a revolutionised CO_2_-utilisation technology sector. This will be further aided by the advancement of the synthetic biology sector, allowing for a new class of responsive, functionalised, and customisable hybrid systems to drive the future of green energy.

## Future perspectives

The microbial conversion of CO_2_ to fuels is promising but the variants of CO_2_-fixation pathways all have different kinetic and ATP requirements. For example, the Calvin cycle is deemed inefficient due to its slow kinetics and high demand for ATP. For this reason, it is essential to design and develop pathways that can support greater carbon fixation. An example of this would be the pyruvate synthase-pyruvate carboxylate-glyoxylate pathway.^123^ Genome-resolved metagenomics has also been used to identify potential enzymes that will show greater efficiency once integrated into CO_2_-fixation pathways. Using metagenomics, Figueroa *et al.* identified genes responsible for phosphite oxidation and CO_2_ reduction within *Candidatus Phosphitivorax anaerolimi* strain Phox-21.^124^ It was discovered that while *Ca. P anaerolimi* contained the genes necessary for Wood–Ljungdahl pathway, the genes for other carbon fixation pathways were absent. Moreover, CO_2_ reduction *via* the Wood–Ljungdahl pathway was not feasible for Phox-21 as certain genes for particular carbonyl steps were missing. In light of these discoveries, Figeuroa *et al.* proposed an approach as to how Phox-21 could reduce CO_2_ to formate before proceeding to formate assimilation pathways. With the rapid development of analytical and biological techniques, carbon fixation may be enhanced to the standards of practical applications.

Expanding the library of microbes may also lead to unfamiliar reaction pathways that can be exploited to give greater STC efficiencies and more energy-dense solar fuels. The metabolic engineering of *Clostridium autoethanogenum* has already led to its large-scale industrial application for the production of ethanol from syngas.^125^ Microorganisms lacking CO_2_-fixing pathways, such as *E. coli* and *S. cerevisiae*, may be transplanted into heterotrophs like *S. oneidensis* and *G. sulfurreducens*. *Pyrococcus furiosus* is also another host candidate and attempts have been made to incorporate CO_2_-fixation pathways into the archaea.^126,127^ The use of thermophile such as *P. furiosus* in hybrid microbial photosystems will be highly beneficial as solar transformations of CO_2_ to volatile biofuels at higher temperatures will become more feasible.

Whilst the study and discovery of new carbon fixation pathways within the microbe is essential for more meaningful CO_2_ transformation, investigations into the energy modules should not be disregarded. As described at the beginning of the review, a lot of energy is required to convert CO_2_ to biofuels due to its high stability. To circumvent this, the study of energy modules has been undertaken regarding semiartificial microbial photosystems. Owing to stoichiometric modelling, and computational analyses we now have a comparative analysis of autotrophic growth and production based on different natural and synthetic CO_2_ fixation pathways and energy uptake.^128,129^ Advances in technology have now allowed for the multi-omics study of not only ATP production, but also electron transfer from semiconductor to microbe and CO_2_ reduction which was carried out by Zhang *et al.*^122^

Understanding the electron transfer between the semiconductor and microbe will also be essential for the future development of hybrid photosystems. Optimisation of the charge transfer processes through genetic engineering can take place, ensuring superior interaction between the biological and semiconductor interface. This in turn will theoretically give us greater STC efficiencies as well as a deeper understanding of how more complex biofuels can be generated.

Metal–organic frameworks (MOF) are becoming an emerging contender for light harvesting. MOFs are composed of transition metal ions that are connected to organic linkers and have crystalline, porous, extended structures. They are structurally tuneable, allowing for the assembly of higher surface areas for the capture of solar energy and its self-assembly into crystalline structures enables the MOF to be computationally analysed.^130,131^ MOFs have shown their capability through successfully harvesting water from desert air with only ambient sunlight as the energy input.^132,133^ For the solar conversion of CO_2_ to biofuels, when interfaced with anaerobic microbes, MOFs have the potential to offer protection from oxygen and ROS, ultimately prolonging the lifetime of the bacteria and ultimately allowing for continuous biofuel production.^134^ In using a zirconium nanocluster-based monolayer to wrap around *M. thermoacetica*, CO_2_ transformation to acetate was able to last approximately 2.5 days whereas, without the MOF protection, CO_2_ fixation was only able to last 1 day.^135^ This was owed to the zirconium nanoclusters present within the MOF as it decomposed the hydrogen peroxide formed on the cell membrane, preventing ROS accumulation. Hydrogels have also been used to shield photosensitised *M. thermoacetica*, extending the viability of the bacterium and increasing acetate production.^136^

Despite the benefits of microbial hybrid photosystems, commercialisation of large-scale production is still in the distant future. The major factors to consider are cost and efficiency, not to mention more research into novel artificial semiconductors that will bolster STC is needed. Hence, fossil fuels are still the most widely used source of fuel production.^137^ However, initiatives such as the Paris Agreement and investments into green energy will help further the advancement of microbial biohybrid photosystems, especially as world leaders initiate the phasing out of fossil fuel industries. Like hybrid photosystems, next-generation materials consisting of smart, functional properties are being heavily invested in. In particular, synthetic biological materials are attracting attention for their potential in the construction of smart living due to their environmental responsiveness, adaptive abilities, self-healing capabilities, and self-regeneration, just to name a few. Several successful engineering efforts have taken place to mimic these biological materials on a large scale. Buildings like the OME, an experimental biological house in the North-East of England which aims to test new, experimental biotechnologies for sustainable living^138^ will help bridge the transition from laboratory to industry, and eventually, to real, everyday life. By monitoring the biological materials' response to the environment, the integration of microbial photosystems into these ‘smart’ properties will become more feasible, alleviating the population's dependency on fossil fuels.

In the 2000s, the invention of the genetic toggle switch first implemented within the plasmid of *E. coli* stimulated the synthetic biology sector and has now reached the fields of material science and engineering.^139^ Rather than relying on bioengineering, the genetic toggle switch is controlled *via* manipulation of the network architecture. Demonstrating that the principles of engineering may be exploited to revolutionize simple cells into living machines, manipulation of the network architecture will enable the control of cell function and maximise STC efficiency. Moreover, by interfacing with inorganic materials such as gold nanoparticles and QDs, numerous environmentally responsive and controllable functional composite materials were developed. As previously mentioned, analysis of these environmental responses and adaption of the cellular functions will be important if the integration of microbial photosystems in buildings is to take place.

In recent years, the ability to transform waste carbon into biomass has become more and more important. Studies in this area include the works of Gleizer *et al.* who reported the engineering of *E. coli* to produce biomass from only CO_2_.^140^ To do this, the glycolysis and pentose-phosphate pathways, were disrupted whilst key enzymes in the Calvin cycle were expressed. A vital step in this study was the introduction of NAD-dependent FDH which would later be used to generate NADH to serve as the reducing power to drive carbon fixation. The strain was left to optimise for CO_2_ fixation by allowing it to adapt and evolve for over 350 days. Genomic sequence analysis confirmed the presence of mutations that regulate gene expression and pathway regulations, overall achieving 100% biomass origin from CO_2_ fixation. Similarly, Gassler and coworkers rewrote the xylulose monophosphate cycle to introduce the Calvin cycle.^141^ Methanol assimilating genes within the peroxisomes of *Pichia pastoris* were deleted and the energy and reducing power were supplied by methanol oxidation. These modifications resulted in the fungi utilising CO_2_ as its sole carbon source, increasing its maximum growth rate on CO_2_ more than two-fold. These studies highlight the importance of not only carbon fixation pathways but also the energy and reducing power on cell growth.

The works of Gleizer and co-workers have set an important milestone towards future works in the sustainable production of biofuels from CO_2_ only, especially in allowing for a more in-depth study of how synthetic CO_2_-fixation pathways can be assimilated into non-native hosts, as well as opening the likelihood of increasing the repertoire of microbes able to undergo solar reduction of CO_2_ where new metabolic pathways may be discovered. The accomplishments of Gleizer and more have stimulated the interdisciplinary field of synthetic biology and the various research currently in motion have demonstrated the great potential of smart materials in the application of sustainable production of biofuels in photohybrid systems.

## Conflicts of interest

There are no conflicts to declare.

## Supplementary Material
